# The circadian regulation of extracellular ATP

**DOI:** 10.1007/s11302-022-09881-3

**Published:** 2022-08-08

**Authors:** Xin Wang, Yu-Ting Dong, Xiu-Ming Hu, Ji-Zhou Zhang, Nan-Rui Shi, Yan-Qin Zuo, Xu Wang

**Affiliations:** School of Acupuncture and Tuina, Chengdu University of Traditional Chinese Medicine, 37 Shi-er Qiao Road, Chengdu, 610075 China

**Keywords:** Purinergic signalling, Extracellular ATP, Circadian rhythm, Suprachiasmatic nucleus, Bladder

## Abstract

Extracellular ATP is a potent signaling molecule released from various cells throughout the body and is intimately involved in the pathophysiological functions of the nervous system and immune system by activating P2 purinergic receptors. Recent increasingly studies showed that extracellular ATP exhibits circadian oscillation with an approximately 24-h periodicity, which participates in regulatory pathways of central oscillator suprachiasmatic nucleus and peripheral oscillator bladder, respectively. Oscillators modulate the protein expression of ATP release channels and ectonucleotidase activity through clock genes; indeed, real-time alterations of ATP release and degradation determine outcomes of temporal character on extracellular ATP rhythm. The regulatory pathways on extracellular ATP rhythm are different in central and peripheral systems. In this review, we summarize the circadian rhythm of extracellular ATP and discuss several circadian regulatory pathways in different organs via ATP release and degradation, to provide a new understanding for purinergic signaling in the regulatory mechanism of circadian rhythm and a potential target to research the circadian regulation of extracellular ATP in other circadian oscillators.

## Introduction

Most organisms evolved endogenous daily physiological and behavioral rhythms to accommodate recurring changes of diurnal environmental, light, and food availability for optimal fitness. These daily rhythms are called circadian (the Latin *circa diem*, about a day) [1]. Since the first discovery of circadian rhythm in cyanobacteria, its existences were observed successively in fungi, bacteria, plants, and animals [1]. Circadian clock not only generates strong rhythm, but also is flexible to adjust its phase in response to environmental perturbation [2]. On the one hand, in molecular level, the core of clock genes is a transcription–translation feedback loop (TTFL) containing both positive components and negative regulatory complexes [3, 4]. The time-dependent variation of its function and interaction endogenously formed a highly rhythmic cycle, which takes approximately 24 h to complete [4]. On the other hand, the phase of circadian rhythm is regulated by environmental cues (termed zeitgebers) via intervening expression and degradation of some specific molecular clock genes; for example, transcriptional inhibitor Timeless (TIM) in *Drosophila* is decomposed under the light condition through the E3 ubiquitin ligase Jetlag (JET) and the photoreceptor protein Cryptochrome (CRY), which was also impaired by JET [5–7]. Thus, intrinsic rhythm of the circadian system is able to keep running with a period of approximately 24 h that is independent of external environment; the phase of circadian rhythm is altered by changed physiology and environment through circadian regulation. The circadian system is the hierarchical network structured by multiple oscillators to synchronize internal rhythms and to coordinate internal clocks with external cycles [8, 9], which consists of three components: input pathways to perceive extrinsic circadian rhythm, a central circadian oscillator, and output pathways leading to the advancement or delay of circadian clock [10]. The suprachiasmatic nucleus (SCN) as central circadian pacemaker sustained to its precision by autoregulatory TTFL, received time-of-day information from the retina to fitted in with external time (entrainment), and conveyed time information to downstream clocks in other peripheral oscillators [1, 9]. Based on the time optimization of physiological processes and circadian regulation, circadian rhythm has an adaptive advantage to fit in behavioral and environmental clock by circadian system.

Purinergic signaling, as a cotransmitter and an extracellular signaling molecule, was firstly proposed by Burnstock [11]. Since the ATP release was discovered in neuron and glial, purinergic signaling has been implicated in the regulation of physiological and pathophysiological activities in the nervous system. Purinergic signal encompasses ATP, ADP, AMP, and ADO, as well as P1 and P2 receptors. In the central nervous system, ATP activates ion channel ligand–gated receptor subtypes (P2X1-P2X7) and G-protein coupled receptor subtypes (P_2Y1_, P_2Y2_, P_2Y4_, P_2Y6_, P_2Y11_, P_2Y12_, P_2Y13_, P_2Y14_), and ADO activates P1 receptor subtypes (A_1_, A_2A_, A_2B_, and A_3_) [12, 13]. The ATP release induced by gentle mechanical stimulation is observed in virtually all cell types and tissues [14]. After release into extracellular fluid and the neuropil, extracellular ATP (eATP) is hydrolyzed to ADP and AMP by the ecto-nucleotide triphosphate diphosphohydrolase family of enzymes (eNTPDases), subsequently dephosphorylated to ADO by ecto-5′-nucleotidase (CD73) [13]. eATP, with its metabolisms ADP-ADO and subsequent receptor-triggered downstream pathways not only regulated the functional activities from cell to tissue, but also participated in the process from physiology to pathology. Pain perception and analgesic effect of ankle arthritis were regulated by acupuncture through eATP [15, 16]. The circadian regulation of hematopoietic stem/progenitor cells release into peripheral blood was coordinated by the eATP-associated pathway [17, 18]. The activation of purinergic receptors participates in the stem cell differentiation and in the neuronal function [19, 20]. In the pathogenesis of colorectal cancer and oral squamous cell carcinoma, eATP mediated cancer cell migration and invasion by the signal-regulated kinase and inflammatory protein [21–24]. The occurrence of cancer is increasingly demonstrated to associate with the chronic disruption of circadian clock [25]. eATP is rapidly hydrolyzed as short-term neurotransmitter in neuromodulation, secretion, and neurotransmission, and plays a role of long-term signaling molecule in cell differentiation, proliferation, and death [26].

There are prominent manifestations of circadian rhythms observed in the daily fluctuation of eATP as neurotransmitter or signaling molecule. In the seminal paper by Yamazaki and Ishida, it was reported that three brain regions, SCN, AHA, and CPu, exhibit circadian rhythms in ATP content (both ATP and eATP) under constant dark conditions [27]. It was shown later that eATP level oscillated in SCN and bladder lumen, as well as in cell cultures from brain regions or bladder. In order to summarize the regulation mechanism of circadian rhythm, this review focuses on eATP rather than intracellular ATP. Time-dependent change of P2 receptors in the SCN and in the hippocampus has been observed at the mRNA and protein expression levels [28, 29]. eATP can not only activate rhythmic P2 receptors, but also provide a source for hydrolysis to ADO. The circadian rhythm of ADO and its associated enzyme CD73 has been deeply studied and applied in cognitive dysfunction, heart disease, and cancer [30, 31]. For extensive evidences of eATP rhythm and regulation, it is noticeable in certain tissues that (1) the detection of eATP level under same conditions might be different at different period, and (2) these differences subsequently induce negative or inverse results in same experiment. Thus, exploring the distribution of eATP rhythm is indispensable for guiding experiment to avoid circadian interference, and summarizing the characters of ATP rhythm in various tissues may provide a potential therapy to relief the circadian aggravation of chronic symptom by ATP-associated intervention. In addition to eATP rhythm, circadian regulation of eATP is directly associated to ATP release pathways and eNTPDases in various oscillators. The occurrence of circadian oscillations in cellular metabolism, electrical activity, and gene expression are accompanied by the generation of the eATP rhythm in an ensemble of SCN region, or even in individual SCN astrocytes [32–35]. Rhythmic fluctuation of eATP levels was observed in the culture of immortalized cell and primary astrocyte in vitro, as well as at SCN and bladder lumen in vivo [32]. The ATP release pathways connexins 43 and NTPDase2 also showed rhythmic expression in bladder and in rat serum [36, 37]. Approximate phase and amplitude were exhibited in the circadian rhythm of neurotransmitters ATP and ADO, which interacted with the regulation of circadian clock [38]. Subsequently, rhythmic eATP has been shown to regulate physiological processes of circadian tissues in the central system. The eATP regulation was involved in the inhibitory transmission of SCN synapses and mechanical pain perception as well as function recovery of astrocyte after metabolic inhibition [39–41]. The electrical activity and arginine vasopressin secretion rhythms of SCN negatively correlate with eATP level [27, 32, 42]; the eATP level altered extracellular H^+^ flux in astrocytes of the hippocampus and cortex [43]. Thus, characterized by stable rhythm and wide participation in multiple system functions, eATP can be used as an important output signal of the circadian rhythm to investigate internal clock and circadian regulation. Although the eATP-associated mechanism in liver tumor and chronic kidney disease [44, 45] overlaps with the increasing rhythmic discoveries at the kidneys, liver, and spleen [46], the regulatory relationship of these overlaps that may form the circadian regulation of eATP is still unclear. In the research of ATP rhythm, there are little result of intracellular ATP, and few articles in the circadian regulation of eATP at other arrhythmic organs and tissues. Because of space restrictions, it has been decided to concentrate on the most relevant articles about the eATP rhythm and its circadian regulation in brain and bladder. In this review, we summarize the ATP rhythm from pacemaker to oscillators, and emphasize the possible implication of circadian regulation on eATP that may modulate various rhythmic diseases as a future intervention.

## The circadian character of eATP rhythm

In the research of circadian pacemaker and oscillators, there were some results about the rhythmic variation of eATP level in central and peripheral systems.

### The circadian character of eATP rhythm in the central system

In the central system, circadian rhythms of eATP level were observed in several brain regions (SCN, anterior hypothalamic area (AHA), caudate putamen (CPu)) and in cell cultures from different brain regions. Various experimental methods including microdialysis and biological and chemiluminescence assay detected about 24-h diurnal change in eATP levels, through the cycle of peak was different among these rhythms (Table 1). Integrating zeitgeber times (ZT) to synchronize the light/dark cycle, the single peak of eATP rhythm mainly presented at night (Table 1) and the low level of eATP slightly fluctuates at other cycles. The occurrence of different peaks on afternoon or at night might be because of (1) the difference of clock axis among circadian pacemaker and subordinate oscillators, and (2) the inevitable experimental affection on the light (rest) cycle during the cultivation of immortalized cells, resulting in the shift of peak cycle. The oscillation can last about 2 to 3 days in short-term organotypic or primary cultures under constant environmental conditions with an approximately 24-h periodicity [47, 48]. This persistent oscillation of eATP in astrocytes might be involved in the cell–cell communication of circadian rhythm in certain brain regions [49]. In addition, the rhythm of extracellular ATP is sensitive to the light changes of external environment: only one change of amplitude increased in the rat SCN brain region after exposed to continuous darkness for 2 days [32]. Thus, eATP rhythm is strong, and sensitively exhibits the influence of entrainment with its amplitude and phase. Noticeable, the paper by Alisa D reported that there is no fluctuation of real time eATP accumulation in the AHA, which is inconsistent with the seminal result in 1994 [27]. That is because the process of microdialysis or the rapid degradation of extracellular ATP itself might dilute the lower concentration of eATP in the AHA compared to in the SCN, eventually flattening the eATP fluctuation. Additionally, as displayed in Table 1, the low amplitude of eATP rhythm observed in organotypic-cultured cell of non-SCN also consists with the result in 1994 [48]. SCN contains numerous astrocytes that also show circadian rhythms both in protein and mRNA levels of the clock gene. In recent research, SCN 2.2 (immortalized rat SCN cells containing 80% astrocytes) and cortical astrocytes are more common to be chosen for the cell cultures in eATP rhythm studies [48]. Forskolin treatment coordinates rhythmic change of clock gene expression and glucose uptake in SCN2.2 cell by enhancing cyclic adenosine monophosphate (cAMP) levels, and finally maintains the endogenous rhythm and pacemaker properties [32, 50]. Asynchronous peaks of astrocyte eATP between SCN and cortex, shown in Table 1, may implicate their regulatory relationship of hierarchical network among master clock pacemaker and subordinate oscillators in the nervous system. However, there are little research of eATP rhythm in other rhythmic brain regions such as the pineal gland, pituitary gland, and olfactory bulb [51], and in certain brain region abundant in astrocyte or other glial cells.

**Table 1:** Rhythmic characteristics of extracellular ATP level. Physiological state: standard lighting condition with 12 h of light and 12 h of dark cycles (LD 12:12). *SCN* suprachiasmatic nucleus, *SCN* cell similar organotypic SCN cultures, *non-SCN cell* organotypic hypothalamic cultures lacking the SCN, TRT-HU1 hTERT-immortalized human urothelial cells, *ZT* zeitgeber time, “-” indicates peak of circadian not detected

	Source	In vivo				In vitro				References
		Specie	Organ/tissue	Experimental techniques	Cycle of peak	Species	Cell	Experimental techniques	Cycle of peak	
Central	SCN	Rat	Tissue	Luciferin-luciferase assay; photon count	6:00–10:00	Rat	SCN2.2 cell	Chemiluminescence assay	14:00–16:00	[27]; [32]
	SCN	Rat	Tissue	Microdialysis; chemiluminescence assay	24:00–02:00	Rat	SCN cell	Bioluminescence assay	24:00–4:00	[32]; [48]
	SCN	Rat	Tissue	Microdialysis; chemiluminescence assay	18:00–20:00	Rat	SCN2.2 cell	Chemiluminescence assay	14:00–16:00	[32]; [32]
	Cortex	-	-	-	-	Rat	Astrocyte	Chemiluminescence assay	14:00–16:00	[32]
	Cortex	-	-	-	-	Mice	Astrocyte	Bioluminescence assay	24:00	[47]
	AHA	Rat	Tissue	Luciferin-luciferase assay; photon count	18:00–22:00	-	-	-	-	[27]
	AHA	-	-	-	-	Rat	AHA cell (without SCN)	Bioluminescence assay	24:00–4:00	[48]
	CPu	Rat	Tissue	Luciferin-luciferase assay; photon count	18:00–22:00	-	-	-	-	[27]
Peripheral	Bladder	Mice	Organ	Luciferin-luciferase assay	1:00	Mice	TRT-HU1 cell	Luciferin-luciferase assay	18:00	[32]; [32]
	Bladder	-	-	-	-	Mice	Urothelial cell	Luciferin-luciferase assay; photon imaging	14:00–18:00	[52]

### The circadian character of eATP rhythm in the peripheral system

In the peripheral system, the classic subordinate oscillator urinary bladder shows strong diurnal rhythm of functional capacity. In the period of bladder fulling, the stretch-released ATP regulates the functional capacity of the bladder by mechanically conducting signals [36]; the content of stretch-released ATP was also observed time-dependent variation in bladder lumen [32]. The eATP release exhibits circadian rhythm with the peak at 14:00–18:00 in primary-cultured urothelial cells and hTERT-immortalized human urothelial cells (TRT-HU1) that the clock genes of these cultures also oscillated at mRNA and protein levels daily [36, 52]. Thus, eATP exhibits the circadian rhythm both in vivo and in vitro as not only a neurotransmitter but also a signaling molecule. The influences of experimental or pathological interventions on circadian clock are presented by the amplitude and phase of ATP rhythm, which may explain the different cycles of peak between cell and organ/tissue. Additionally, the retina, as an input pathway that conveys the environmental information of light/dark cycle to central nervous, also displays circadian rhythm of overall ATP content with a peak at early night [53]. The eATP released from synaptic vesicles or Muller glial cells supports the main source of extracellular adenosine (ADO), which might participate in the regulation of light/dark adaptation on ADO [53, 54]. However, there are few reports about diurnal change of eATP in the retina, as well as other rhythmic tissues.

## The circadian regulation of eATP release

### The circadian regulation of eATP release by clock genes

**Fig. 1:**
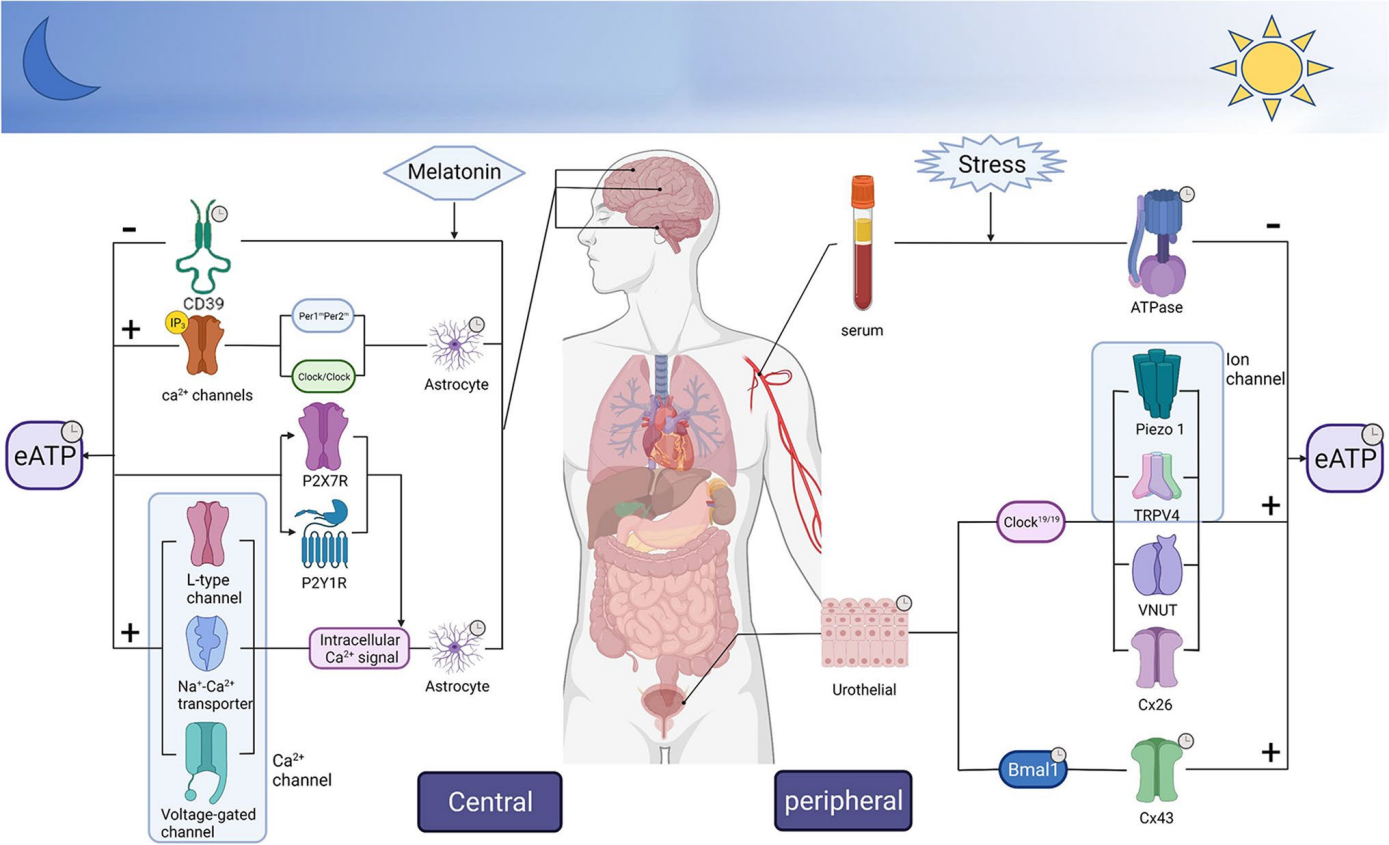
The regulation on pathway of eATP rhythm in central (prosencephalon, cortex, and SCN) and peripheral (serum and urothelium) systems. Molecular clocks in central and peripheral systems drive eATP rhythm by specific release pathways and degradation pathways. Regulation that impacts any rhythmic eATP release (e.g., Ca^2+^ channels, icon channel, exocytosis, or connexins) represents latent targets for controlling circadian pathways by molecular clocks (Bmal1, Clock, and Per) at organs and tissues. Shown are on the top the degradation pathway regulated by melatonin in the prosencephalon through CD39 and mediated by restraint stress in serum via ATPase. Upper right clock of icon denotes its own circadian rhythm. + indicates release pathway;—indicates degradation pathway

The diurnal effect from external environment or internal intervention on the eATP rhythm almost acts on the eATP release or the eATP degradation. As shown in Table 2, the alteration of eATP rhythm was various after the intervention of light environment and clock gene. Under the lighting condition with constant darkness (DD), the eATP rhythm in the rat SCN displayed a higher peak during the middle of the subjective night [32]. The rhythm of eATP release was observed that lower diurnal fluctuation and sharply decreasing release both in *Clock* mutant astrocytes and *Per* mutant astrocytes [47], whereas primary-cultured urothelial from Clock mutant mice [52] maintained a peak level approximate to wild-type urothelial in the diurnal variation of extracellular ATP release (Fig. 1). Conversely, the ATP release of urinary bladder lumen at ZT19 was reduced in arrhythmic *Bmal1*-knockout mice, which was consistent with the amplitude of eATP rhythm in TRT-HU1 cells blunted by *Bmal1* knockdown [36]. Similar interventions in circadian rhythm lead to different or even opposite alteration of eATP rhythm, and indicate that clock genes might mediate different pathways of eATP release or degradation. Thus, it is crucial to investigate the eATP release pathway and its hydrolase to research the circadian regulation of ATP rhythm by clock genes.

**Table 2:** The effects of intervention on extracellular ATP rhythm. *AHA* anterior hypothalamic area, *CPu* caudate putamen, *DD* constant darkness, *dnSNARE* blocking vesicular gliotransmission, *VIPP* impairing IP3-dependent calcium signaling pathways, *Cx43* Connexin 43, *VNUT* vesicle nucleotide transporter mediates ATP secretion, *Piezo1* piezo-type mechanosensitive ion channel component 1, *TRPV4* transient receptor potential cation channel subfamily V member 4, *Cx26* Connexin26. + / − exhibits circadian rhythm or no rhythm. NA indicates amplitude/phase/period of circadian not applied; ↑or ↓ indicates amplitude/phase of circadian increases or reduction,; ± indicates amplitude/phase/period has no significant change

Species	Tissue/cell	Experimental techniques	Intervention		Variation on rhythm				References
					Rhythm (+ / −)	Amplitude	Phase	Period	
Rat	SCN tissue	Microdialysis; chemiluminescence assay	Light environment	DD	+	↑	±	±	[32]
Mice	Cortical astrocyte	Bioluminescence recording; immunocytochemistry	Clock gene	*Per1Per2* double mutant	+	↓	↓	↓	[47]
Mice	Cortical astrocyte	Bioluminescence recording; immunocytochemistry		*Clock/Clock* mutant	+	↑	↓	±	[47]
Mice	urothelial cell	Luciferin-luciferase assay; photon imaging		*Clock* mutant	−	↓	↑	NA	[52]
Mice	TRT-HU1 cell	Rtq-PCR; bioluminescence recording; luciferin-luciferase assay		*Bmal1* knockdown	+	↓	±	NA	[36]
Mice	Cortical astrocyte	Bioluminescence recording; immunocytochemistry	Vesicular release	dnSNARE	+	±	±	±	[47]
Mice	TRT-HU1 cell	Rtq-PCR; bioluminescence recording; luciferin-luciferase assay	Connexin hemichannel	Cx43 knockdown	−	↓	NA	NA	[36]
Mice	TERT-NHUC cell	Rtq-PCR; bioluminescence recording; luciferin-luciferase assay		Cx43 overexpression	−	↑	NA	NA	[36]
Mice	Urothelial cell	Luciferin-luciferase assay; photon imaging		Cx26 knockdown	+	↓	±	NA	[52]
Rat	SCN cell	Bioluminescence assay; immunohistochemistry	Pannexin plasma membrane channel	Inhibition of panneixn-1 hemichannels carbenoxolone	+	±	±	±	[48]
Mice	Cortical astrocyte	Bioluminescence recording; immunocytochemistry	Calcium signaling pathway	VIPP	−	↓	↓	NA	[47]
Rat	SCN cell	Bioluminescence assay; immunohistochemistry		Selective inhibition of mitochondrial Na + -Ca2 + exchange transporter CGP37157	−	↓	±	NA	[48]
Rat	SCN cell	Bioluminescence assay; immunohistochemistry		The Ltype Ca2 + channel inhibitor nifedipine	−	↓	±	NA	[48]
Rat	SCN cell	Bioluminescence assay; immunohistochemistry		Voltage-gated Ca2 + channel blocker La3 +	−	↓	±	NA	[48]
Rat	SCN cell	Bioluminescence assay; immunohistochemistry	Purinergic signaling pathway	Selective P2X7R antagonist AZ10606120	−	↓	±	NA	[48]
Rat	SCN cell	Bioluminescence assay; immunohistochemistry		Selective P2X7R antagonist A438079	−	↓	±	NA	[48]
Rat	SCN cell	Bioluminescence assay; immunohistochemistry		Classical P2X7R antagonist BBG	−	↓	±	NA	[48]
Rat	SCN cell	Bioluminescence assay; immunohistochemistry		Positive allosteric modulator of P2X7R GW791343	+	±	↑	NA	[48]
Rat	SCN cell	Bioluminescence assay; immunohistochemistry		Nonselective P2 receptor antagonist PPADS	−	↓	±	NA	[48]
Rat	SCN cell	Bioluminescence assay; immunohistochemistry		P2X4R-selective negative allosteric modulator 5-BDBD	+	±	±	±	[48]
Rat	SCN cell	Bioluminescence assay; immunohistochemistry		P2Y1R-selective antagonist MRS2179	−	↓	↓	NA	[48]
Rat	SCN cell	Bioluminescence assay; immunohistochemistry		P2Y1R-selective agonist MRS2365	+	↑	↑	NA	[48]
Rat	SCN cell	Bioluminescence assay; immunohistochemistry		P2Y2R-selective agonist MRS2768	+	↑	↑	NA	[48]

### The circadian regulation of eATP release by vesicular pathway

eATP level is directly mediated by associated release pathways and the ecto-nucleotide triphosphate diphosphohydrolase family of enzymes, and the 24-h temporal eATP rhythm is formed by the real-time ratio of its release and hydrolysis. In general, there are two release mechanisms of intracellular ATP: vesicular and non-vesicular pathways [14]. VNUT (a member of the solute carrier 17 family of ion transporters) transferred cytosolic ATP into secretory granules, and contributed to the eATP release as a vesicular nucleotide transporter [55]. Knocking down VNUT by small interfering RNA (siRNA) reduced KCl-associated exocytosis of ATP [55]. Importantly, the stretch-released ATP at 12 h was significantly attenuated in the time-dependent variation of eATP in VNUT-knockout urothelial cell [52]. In in situ hybridization studies, extensive VNUT transcripts are observed in many brain regions, especially in the hippocampus that has shown abundant purinergic neurotransmission [56], where it might exhibit undetected eATP rhythm. Moreover, the exocytosis release of ATP was also coordinately regulated by soluble N-ethyl maleimide–sensitive factor attachment protein receptor (SNARE) family of proteins [57]. Blocking vesicular release from cortical astrocyte by dominant-negative (dn) SNARE has no significant difference in the circadian rhythm of ATP release [48]. Thus, the circadian regulation of exocytotic ATP release is different between cortical astrocyte and urothelial cell.

### The circadian regulation of eATP release by non-vesicular pathways

In addition to the classical exocytotic release, non-vesicular mechanism of cellular ATP release has been demonstrated in various oscillators, which contains five groups of release channels: pannexin-1, calcium homeostasis modulator 1, connexin hemichannels, volume-regulated anion channels, and maxi-anion channels [58]. There are accumulating evidences that ATP release was mediated by P2X7 [59–61] that associated with not only pannexin-1, but also unknown protein pathways [62–65].

#### The circadian regulation of eATP release by pannexin-1

The pannexin (PANX) is the well-demonstrated non-junctional membrane channels that are not evolutionarily connected to gap junction-forming connexins [66, 67]. Among the pannexin gene family, PAN1 has been proved to regulate extracellular autocrine and paracrine purinergic signaling as ATP release channel; it is expressed in most cells without vesicular ATP release. The ATP release site of polar secretion presents PAN1 with the absence of connexin [68, 69]. Carbenoxolone (Cbx, a gap junction and hemichannel inhibitor) significantly flattened the diurnal fluctuation of mechanical-induced ATP release in immortalized urothelial cell [36], but had a partial inhibitory effect on the amplitude of circadian eATP rhythm in SCN cell [48]. There must be subtle differences of circadian regulation in eATP release pathways from different pacemakers/oscillators. PANX1 presents two kinds of open channel conformations through different activation modes: a large-conductance, non-selective, ATP-permeable conformation and an intermediate-conductance, anion-selective, ATP-impermeable conformation [70]. eATP can activate PANX1 to the transformation of ATP permeability conformation via direct excitement of P2X7 or through Ca^2+^-dependent P_2YR_ stimulation, subsequently contributing to ATP-induced ATP release [70, 71]. The positive feedback loop is ended by the inhibition of its ATP permeability conformation by the high concentration of eATP. Thus, this positive feedback loop of eATP release on PAN1 might be associated with the amplitude of eATP rhythm as the factor that produces the peak of eATP rhythm, consistent with the results of PAN1 or P2 receptors on Table 2. However, the electrophysiological fingerprint of PANX1 activation mode inducing the ATP-impermeable conformation remains unclear.

#### The circadian regulation of eATP release by calcium homeostasis modulator 1

The calcium homeostasis modulator 1 (CALHM1) is the pore-forming subunit of a plasma membrane voltage-gated ion channel similar to the structure of connexin and pannexin [72–74]. The rhythm of ATP release oscillated in SCN astrocyte independent of neuronal cell populations [32], and its release was induced by transient increase of Ca^2+^ [75]. Importantly, there is an inverse phase relationship between the circadian rhythm of intracellular Ca^2+^ concentration and the eATP rhythm [76]. CALHM1 is closed at the resting membrane voltage of physiological extracellular Ca^2+^ concentration and opened at strong depolarization and more negative membrane voltages [47, 73]; the peak of intracellular Ca^2+^ rhythm is not enough to trigger calcium-dependent ATP release [77]. This character of CALHM1 may explain the inverse phase relationship of circadian rhythm between intracellular Ca^2+^ concentration and the eATP accumulation. In cortex astrocyte, the daily circadian of ATP release depends on IP_3_ signaling-associated Ca^2+^ channel and clock genes (*Clock* and *Per*). In SCN astrocyte, three Ca^2+^ channels, selective inhibition of mitochondrial Na^+^-Ca^2+^ exchange transporter, L-type Ca^2+^ channel inhibitor, and non-selective voltage-gated Ca^2+^ channel blocker, were demonstrated to abolish the circadian rhythm of ATP release and inhibit the cumulated ATP release in SCN cell (Fig. 1) [45]. The expression of CALHM1 was detected both in hippocampal and cortical neurons, which inferred that CALHM1 was involved in the circadian regulation of extracellular ATP by modulating the expression of P2X and P_2Y_ [28, 73]. The ATP release rhythm of cortical astrocyte was abolished by high expression of enzyme that terminates IP3 signaling (VIPP) through impairment of IP3-dependent calcium signaling pathways (Fig. 1) [47]. These data provide considerable evidences that astrocyte activates calcium signaling pathways to mediate the release of ATP rhythms [47]. As shown in Table 2, P2X7 and P_2Y1_ on astrocyte were involved in the oscillation of eATP circadian rhythm: the amplitude of this rhythm was markedly increased by P_2Y1_-selective agonist, P_2Y2_-selective agonist, and positive allosteric modulator of P2X7; the phase of eATP rhythm had a significant increase after two treatments of P_2Y_ antagonists. Single cell calcium measurement showed that ATP- or BzATP-induced increasements of intracellular Ca^2+^ concentration are inhibited by selective blockers of P2X7 and P_2Y1_ [48]. To sum up, P2X7 and P_2Y1_ positively promoted eATP release by elevating intracellular calcium concentration (Fig. 1), which may be an important element to generate the steep peak of eATP rhythm.

#### The circadian regulation of eATP release by connexins

Connexin subunits are the structural component of vertebrate gap junction channels with more than 20 kinds of molecular weight; connexins can be divided into intercellular channels (gap junctions) and plasma membrane inserted hemichannels (undocked hemichannels) in various mammalian tissues [58, 78, 79]. Main connexin hemichannels are activated by positive membrane potentials; the gating of positive membrane potential modulates connexin hemichannel function via phosphorylation and the redox state in physiological or pathological conditions [80–83]. Although more than 20 connexin subunits confer different permeability property to hemichannels through different single-channel conductance, charge selectivities, and tracer permeability, the pores of functional connexin channels are commonly wide enough to be an access of a variety of small and soluble molecules. ATP is a significant part of small signaling molecular permeations released by connexin hemichannels pores into the extracellular space [58].

Connexin 43 (Cx43 named after its predicted molecular weight) is a major gap junction channel to release ATP in urothelium [84], and Cx43 also showed circadian rhythm at mRNA level and at protein expression in vivo [36]. The rhythm of Cx43 protein expression peaks at ZT07 and falls to the trough at ZT19, which was in accordance with the cycle of peak and trough on ATP rhythm in the bladder lumen [36]. The ATP concentration derived from mechanically induced release was significantly decreased by Cx43-knockdown (Table 2), and this concentration had a considerable increase in Cx43-overexpressing immortalized urothelial cells (TERT-NHUC cells). Furthermore, GAP19 (specific Cx43 hemichannel blocker) efficiently inhibited the mechanically induced ATP release in TERT-NHUC cells and the ATP release caused by bladder distention in mice at ZT19 [36]. Therefore, the daily variation of Cx43 may directly contribute to the circadian change of eATP release in mice urothelium. In Bmal1-knockout mice, the circadian rhythm disappeared both in the mechanically induced ATP release and in Cx43 protein expression, which suggests that BMAL1 exhibits circadian rhythm of mouse bladder through direct regulation of ATP release by Cx43 (Fig. 1). Synchronous circadian oscillations between ATP release and Cx43 expression also supply a valuable reference to further searching for other unclear circadian regulation of eATP. The time-dependent change of stretch-released ATP photons was not observed for Clock^19/19^ mice compared to rhythmic WT mice, and the peak of stretch-released ATP rhythm at 12:00 was significantly attenuated in Cx26-knockdown primary urothelial cell [52]. Therefore, Clock19/19 regulates the circadian rhythm of eATP in urothelial cell that may also depend on the ATP release channel Cx26 (Fig. 1).

#### The circadian regulation of eATP release by ion channels

The activation and conformational transformation of connexin hemichannels are robustly regulated by the extracellular Ca^2+^ concentration [85, 86]. In the urinary bladder, the significant inhibitory effect of nonspecific CALHM1 blocker on ATP release was induced by the hypotonic stress and the depletion of Ca^2+^ [87]. The knockdown of other ion channels, such as piezo-type mechanosensitive ion channel component 1 (Piezo1) and transient receptor potential cation channel subfamily V member 4 (TRPV4), showed a significant inhibitory effect on stretch-released ATP levels at 12 h in urothelial cell [52]. To sum up, clock genes might exhibit circadian rhythm of eATP through different ATP release pathways in different pacemakers or oscillators. In addition, investigating the level on two time points (the peak and the trough) might be not enough to exhibit the circadian rhythm of eATP and to demonstrate the impact of intervention on eATP release.

## The circadian regulation of eATP degradation

Extracellular nucleotides and nucleosides are degraded by a cell surface–located group of enzymes called ectonucleotidase. Ectonucleotidase comprises the nucleoside triphosphate diphosphohydrolase enzyme family (NTPDases) and ecto-5′-nucleotidase, which has been demonstrated to widely distribute in the vascular system and nervous system [88]. NTPDases and 5′-nucleotidase mediate purinergic signaling through the ligand availability to P1 and P2 receptors expressed in various tissues, then regulate the duration of extracellular ATP, ADP, and adenosine [89]. NTPDases contain NTPDases 4–7 that has been observed its intracellular localization, as well as NTPDases 1, 2, 3, and 8 located on the cell surface as an extracellular catalytic site [90]. NTPDase1 was also called CD39 by immunologists and enzymologists [91]. In the mRNA detection of NTPDases on the prosencephalon, NTPDase1 was inspected in the striatum, hypophysial pars tuberalis (PT), and the anteroventral thalamic nuclei (AV), and the signal of NTPDase2 was probed in the hippocampal dentate gyrus (DG) and the medial thalamus, as well as the high expression of NTPDase3 was observed in the striatum, paraventricular thalamic nucleus, and anterodorsal thalamic nucleus [92]. These results from various brain regions may contribute to the subsequent research of eATP rhythm in more regions and of the circadian network regulation. In the bladder, the high expression of NTPDase1 was observed in endothelial cells, NTPDase2 (also known as ATPase) uniquely distributed in the deeper layer of detrusor, and the localization of NTPDase3 partially occurred in the cell membrane of intermediate and basal cells [93]. Differential expressions of NTPDases in different pacemakers/oscillators may provide an explanation to eATP temporal pattern for maintaining low-level fluctuation and for quickly dropping to the trough.

According to the 24-h temporal pattern of ATPase in blood serum, ATPase activity presented diurnal changes with the peak in dark (activity) cycle and the trough on light (rest) cycle (Fig. 1) [37], consistent with the time-dependent variations of NTPDase 1–3 expression in the prosencephalon: the mRNA levels of NTPDase 1–3 peaked during the active phase (CT18) [92]. This might explain the drastic reduction of eATP level on the late dark phase and the occurrence of ADO-peak during late dark cycle. In addition, the activation of NTPDases depends on a certain concentration of Ca^2+^ or Mg^2+^ [94]; Ca^2+^-induced ATP release and active NTPDases might lead to increased release and sufficient hydrolysis during early dark phase, consequently contributing to the occurrence of the peak on ADO rhythm after hydrolysis. The mRNA expression of NTPDase1 was significantly inhibited in melatonin-deficient mice during light (rest) cycle, and elastically rose back to same peak as in melatonin-proficient mice during dark (activity) cycle [94]. After hormonal intervention in the dark phase of NTPDases peak, only physiological-level epinephrine promoted the hydrolysis of adenine nucleotides, rather than melatonin or dexamethasone at physiological-level [37]. Thus, the regulation of NTPDase1 rhythm in the prosencephalon may be limited by melatonin when its concentration is higher than physiological level (Fig. 1).

There are few articles about the circadian regulation between eATP degradation and clock genes (internal circadian). In the research of circadian rhythm intervened by external environment, restraint stress significantly depressed the activity of serum ATPase at night (Fig. 1) [95]. The circadian rhythm of eATP was not only mediated by clock genes, but also affected by external environment. In addition to restraint stress, there are many methods to make rhythm disorder model by light condition, sleep, and temperature. However, the circadian regulation of external intervention on eATP rhythm is not clear.

The clock gene was also transiently influenced by ATP in microglial cells. After 1 mM ATP exposure to microglial cell line BV-2 cells or murine microglia, the mRNA expression of Per1 transiently increased in a manner dependent on P2X7 activity, and no variation in other clock genes (Per3, Dec1-2, Cry1-2, Dbp, Bmal1, and Npas2) [96]. The role of exogenous ATP suggests that the pathological change, like inflammation with high concentration of ATP, might induce the endogenous rhythm disorder, and subsequently exhibit the diurnal variation of inflammatory symptoms.

## Conclusion and perspectives

It is clear from this review that eATP exhibits strong circadian rhythm as a cotransmitter released from nerves/astrocytes and as an autocrine or paracrine messenger released from urothelial cells. Next, eATP release pathways and NTPDases that directly affect the circadian rhythm of eATP level are being elucidated, like calcium signaling pathway, panx1 and Cx43, and NTPDase1 and ATPase in Fig. 1. Understanding how release and degradation of eATP rhythm work together has supported a direct view of the relationship between clock genes and eATP rhythm, in particular the peak of eATP rhythm. These advances of circadian regulation on eATP are showing us how pacemakers/oscillators from different tissues/cells exhibit synchronous eATP rhythm via different regulatory pathway, and how oscillators output different environmental/internal stimuli to eATP rhythm. However, the downstream mechanisms of regulatory pathways also interact, which increases the complexity of mechanism research. Finally, investigating the circadian character and the intricate regulation of eATP rhythm will allow us to modify the time for experimental detection to reduce diurnal interference, and to develop time-efficient treatments.

## Data Availability

Not applicable.
